# 1-[2-(4-Bromo­benz­yloxy)-2-phenyl­ethyl]-1*H*-1,2,4-triazole

**DOI:** 10.1107/S1600536808027748

**Published:** 2008-09-13

**Authors:** Özden Özel Güven, Hakan Tahtacı, Simon J. Coles, Tuncer Hökelek

**Affiliations:** a Zonguldak Karaelmas University, Department of Chemistry, 67100, Zonguldak, Turkey; b Southampton University, Department of Chemistry, Southampton, SO17 1BJ, England; c Hacettepe University, Department of Physics, 06800 Beytepe, Ankara, Turkey

## Abstract

In the mol­ecule of the title compound, C_17_H_16_BrN_3_O, the triazole ring is oriented at dihedral angles of 6.14 (9)° and 82.08 (9)°, respectively, with respect to the phenyl and bromo­benzene rings. The dihedral angle between the bromo­benzene and phenyl rings is 87.28 (7)°. The intra­molecular C—H⋯O hydrogen bond results in the formation of a planar five-membered ring, which is oriented at a dihedral angle of 0.13 (6)° with respect to the bromo­benzene ring. There is an inter­molecular C—H⋯π contact between a methyl­ene group and the bromo­benzene ring.

## Related literature

For general backgroud, see: Paulvannan *et al.* (2001 ▶); Godefroi *et al.* (1969 ▶); Özel Güven *et al.* (2007*a* ▶,*b* ▶); Wahbi *et al.* (1995 ▶). For related literature, see: Peeters *et al.* (1979 ▶); Freer *et al.* (1986 ▶); Özel Güven *et al.* (2008*a* ▶,*b* ▶,*c* ▶,*d* ▶); Özel Güven, Tahtacı *et al.* (2008 ▶).
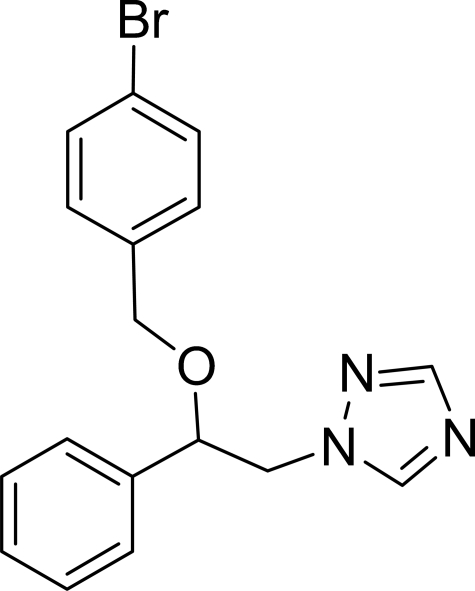


## Experimental

### 

#### Crystal data

C_17_H_16_BrN_3_O
*M* *_r_* = 358.24
Monoclinic, 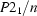

*a* = 10.2070 (2) Å
*b* = 13.7948 (3) Å
*c* = 11.4007 (2) Å
β = 100.317 (1)°
*V* = 1579.31 (5) Å^3^
*Z* = 4
Mo *K*α radiation
μ = 2.61 mm^−1^
*T* = 120 (2) K
0.38 × 0.30 × 0.20 mm

#### Data collection

Bruker–Nonius Kappa CCD diffractometer
Absorption correction: multi-scan (*SADABS*; Sheldrick, 2007 ▶) *T*_min_ = 0.400, *T*_max_ = 0.590
18914 measured reflections
3617 independent reflections
2901 reflections with *I* > 2σ(*I*)
*R*_int_ = 0.051

#### Refinement

*R*[*F* ^2^ > 2σ(*F* ^2^)] = 0.036
*wR*(*F* ^2^) = 0.082
*S* = 1.05
3617 reflections
199 parameters
H-atom parameters constrained
Δρ_max_ = 0.28 e Å^−3^
Δρ_min_ = −0.53 e Å^−3^

### 

Data collection: *COLLECT* (Hooft, 1998 ▶); cell refinement: *DENZO* (Otwinowski & Minor, 1997 ▶) and *COLLECT*; data reduction: *DENZO* and *COLLECT*; program(s) used to solve structure: *SHELXS97* (Sheldrick, 2008 ▶); program(s) used to refine structure: *SHELXL97* (Sheldrick, 2008 ▶); molecular graphics: *ORTEP-3 for Windows* (Farrugia, 1997 ▶); software used to prepare material for publication: *WinGX* publication routines (Farrugia, 1999 ▶) and *PLATON* (Spek, 2003 ▶).

## Supplementary Material

Crystal structure: contains datablocks I, global. DOI: 10.1107/S1600536808027748/xu2450sup1.cif

Structure factors: contains datablocks I. DOI: 10.1107/S1600536808027748/xu2450Isup2.hkl

Additional supplementary materials:  crystallographic information; 3D view; checkCIF report

## Figures and Tables

**Table 1:** Hydrogen-bond geometry (Å, °)

*D*—H⋯*A*	*D*—H	H⋯*A*	*D*⋯*A*	*D*—H⋯*A*
C13—H13⋯O	0.93	2.37	2.723 (3)	102
C11—H11*A*⋯*Cg*3^i^	0.97	2.84	3.687 (2)	147

Symmetry code: (i) 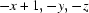. *Cg*3 is the centroid of the C12–C17 ring.

**Fig. 1.**
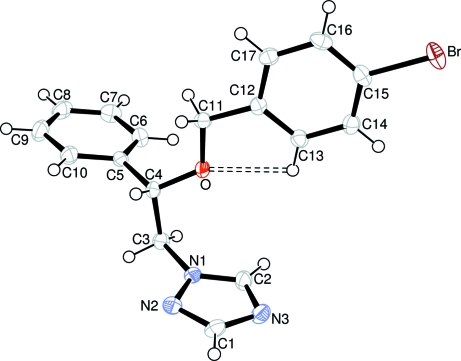
The molecular structure of the title molecule with the atom-numbering scheme. Displacement ellipsoids are drawn at the 50% probability level.

## supplementary crystallographic information

### Comment

In recent years, among antifungal agents, azole derivatives still have an important place in the class of systemic antifungal drugs. 1,2,4-Triazoles are biologically interesting molecules and their chemistry is receiving considerable attention due to antihypertensive, antifungal and antibacterial properties (Paulvannan *et al.*, 2001). Similar structures possessing imidazole ring such as miconazole, econazole and sulconazole have been developed for clinical uses as antifungal agents (Godefroi *et al.*, 1969) and also similar structures possessing benzimidazole ring have been reported to show antibacterial activity more than antifungal activity (Özel Güven *et al.*, 2007*a*,b). Antifungal activity of aromatic ethers possessing 1,2,4-triazole ring have been reported (Wahbi *et al.*, 1995). The crystal structures of miconazole (Peeters *et al.*, 1979), econazole (Freer *et al.*, 1986) and similar structures possessing benzimidazole ring (Özel Güven *et al.*, 2008*a*,b,c,d) have been reported, previously, and now we report herein the crystal structure of the title 1,2,4-triazole ring substituted ether structure.

In the molecule of the title compound (Fig. 1) the bond lengths and angles are generally within normal ranges. The planar triazole ring is oriented with respect to the phenyl and bromobenzene rings at dihedral angles of 6.14 (9)° and 82.08 (9)°, respectively. Atoms C3, C4 and C11 are 0.114 (2), -0.076 (2) and 0.015 (2) Å away from the ring planes of the corresponding triazole, phenyl and bromobenzene, respectively. So, they are nearly coplanar with the adjacent rings. The bromobenzene ring is oriented with respect to the phenyl ring at a dihedral angle of 87.28 (7)°. The intramolecular C—H···O hydrogen bond results in the formation of a planar five-membered ring (O/H13/C11—C13), which is oriented with respect to bromobenzene ring at a dihedral angle of 0.13 (6)°. So, they are coplanar.

In the crystal structure, the molecules are elongated along [010], and stacked along the *c* axis. There is a C—H···π contact (Table 1) between the methylene group and the bromobenzene ring.

### Experimental

The title compound was synthesized by the reaction of 1-phenyl-2-(1*H*-1,2,4 -triazol-1-yl)ethanol (Özel Güven, Tahtacı *et al.*, 2008) with NaH and appropriate benzyl halide. To the solution of alcohol (300 mg, 1.586 mmol) in DMF (4 ml) was added NaH (63 mg, 1.586 mmol) in small fractions. The appropriate benzyl halide (396 mg, 1.586 mmol) was added dropwise. The mixture was stirred at room temperature for 3 h, and excess hydride was decomposed with methyl alcohol (5 ml). After evaporation to dryness under reduced pressure, the crude residue was suspended with water and extracted with methylene chloride. The organic layer was dried over anhydrous sodium sulfate and then evaporated to dryness. The crude residue was purified by chromatography on a silica-gel column using chloroform as eluent. Crystals suitable for X-ray analysis were obtained by the recrystallization of the ether from ethyl acetate (yield; 368 mg, 65%).

### Refinement

H atoms were positioned geometrically, with C—H = 0.93, 0.98 and 0.97 Å for aromatic, methine and methylene H, respectively, and constrained to ride on their parent atoms with *U*_iso_(H) = 1.2*U*_eq_(C).

### Crystal data

**Table:**

C_17_H_16_BrN_3_O	*F*(000) = 728
*M**_r_* = 358.24	*D*_x_ = 1.507 Mg m^−^^3^
Monoclinic, *P*2_1_/*n*	Mo *K*α radiation, λ = 0.71073 Å
Hall symbol: -P 2yn	Cell parameters from 3224 reflections
*a* = 10.2070 (2) Å	θ = 2.9–27.5°
*b* = 13.7948 (3) Å	µ = 2.61 mm^−^^1^
*c* = 11.4007 (2) Å	*T* = 120 K
β = 100.317 (1)°	Block, colorless
*V* = 1579.31 (5) Å^3^	0.38 × 0.30 × 0.20 mm
*Z* = 4	

### Data collection

**Table:**

Bruker–Nonius Kappa CCD diffractometer	3617 independent reflections
Radiation source: fine-focus sealed tube	2901 reflections with *I* > 2σ(*I*)
graphite	*R*_int_ = 0.051
Detector resolution: 9.091 pixels mm^-1^	θ_max_ = 27.5°, θ_min_ = 3.3°
φ & ω scans	*h* = −13→12
Absorption correction: multi-scan (*SADABS*; Sheldrick, 2007)	*k* = −17→17
*T*_min_ = 0.400, *T*_max_ = 0.590	*l* = −14→14
18914 measured reflections	

### Refinement

**Table:**

Refinement on *F*^2^	Primary atom site location: structure-invariant direct methods
Least-squares matrix: full	Secondary atom site location: difference Fourier map
*R*[*F*^2^ > 2σ(*F*^2^)] = 0.037	Hydrogen site location: inferred from neighbouring sites
*wR*(*F*^2^) = 0.082	H-atom parameters constrained
*S* = 1.05	*w* = 1/[σ^2^(*F*_o_^2^) + (0.0282*P*)^2^ + 1.0904*P*] where *P* = (*F*_o_^2^ + 2*F*_c_^2^)/3
3617 reflections	(Δ/σ)_max_ < 0.001
199 parameters	Δρ_max_ = 0.28 e Å^−^^3^
0 restraints	Δρ_min_ = −0.53 e Å^−^^3^

### Special details

**Table:**

Geometry. All e.s.d.'s (except the e.s.d. in the dihedral angle between two l.s. planes) are estimated using the full covariance matrix. The cell e.s.d.'s are taken into account individually in the estimation of e.s.d.'s in distances, angles and torsion angles; correlations between e.s.d.'s in cell parameters are only used when they are defined by crystal symmetry. An approximate (isotropic) treatment of cell e.s.d.'s is used for estimating e.s.d.'s involving l.s. planes.
Refinement. Refinement of *F*^2^ against ALL reflections. The weighted *R*-factor *wR* and goodness of fit *S* are based on *F*^2^, conventional *R*-factors *R* are based on *F*, with *F* set to zero for negative *F*^2^. The threshold expression of *F*^2^ > σ(*F*^2^) is used only for calculating *R*-factors(gt) *etc*. and is not relevant to the choice of reflections for refinement. *R*-factors based on *F*^2^ are statistically about twice as large as those based on *F*, and *R*- factors based on ALL data will be even larger.

### Fractional atomic coordinates and isotropic or equivalent isotropic displacement parameters (Å^2^)

**Table:**

	*x*	*y*	*z*	*U*_iso_*/*U*_eq_	
Br	0.01308 (3)	0.204749 (18)	0.24099 (2)	0.03377 (10)	
O	0.23831 (15)	0.48903 (11)	0.72395 (13)	0.0219 (3)	
N1	0.43625 (18)	0.62535 (13)	0.70844 (15)	0.0209 (4)	
N2	0.4116 (2)	0.71851 (14)	0.67091 (18)	0.0262 (4)	
N3	0.4832 (2)	0.62873 (15)	0.52812 (17)	0.0264 (4)	
C1	0.4421 (2)	0.71587 (17)	0.5630 (2)	0.0260 (5)	
H1	0.4357	0.7703	0.5141	0.031*	
C2	0.4777 (2)	0.57391 (17)	0.62277 (19)	0.0250 (5)	
H2	0.4998	0.5085	0.6287	0.030*	
C3	0.4052 (2)	0.59254 (17)	0.82170 (18)	0.0230 (5)	
H3A	0.4249	0.6441	0.8800	0.028*	
H3B	0.4615	0.5377	0.8500	0.028*	
C4	0.2599 (2)	0.56308 (16)	0.81142 (18)	0.0209 (5)	
H4	0.2028	0.6187	0.7839	0.025*	
C5	0.2348 (2)	0.53134 (16)	0.93291 (18)	0.0203 (5)	
C6	0.2718 (2)	0.43995 (17)	0.9773 (2)	0.0264 (5)	
H6	0.3090	0.3959	0.9308	0.032*	
C7	0.2536 (2)	0.41385 (19)	1.0911 (2)	0.0292 (5)	
H7	0.2780	0.3523	1.1202	0.035*	
C8	0.1994 (3)	0.47886 (19)	1.1609 (2)	0.0306 (6)	
H8	0.1876	0.4614	1.2371	0.037*	
C9	0.1627 (3)	0.57037 (19)	1.1170 (2)	0.0323 (6)	
H9	0.1269	0.6147	1.1640	0.039*	
C10	0.1791 (2)	0.59612 (18)	1.0031 (2)	0.0263 (5)	
H10	0.1527	0.6572	0.9734	0.032*	
C11	0.1027 (2)	0.45894 (17)	0.69514 (19)	0.0220 (5)	
H11A	0.0452	0.5153	0.6806	0.026*	
H11B	0.0788	0.4230	0.7613	0.026*	
C12	0.0843 (2)	0.39583 (16)	0.58547 (18)	0.0199 (5)	
C13	0.1877 (2)	0.37627 (17)	0.52494 (19)	0.0224 (5)	
H13	0.2718	0.4021	0.5525	0.027*	
C14	0.1669 (2)	0.31831 (17)	0.42329 (19)	0.0240 (5)	
H14	0.2367	0.3050	0.3833	0.029*	
C15	0.0416 (2)	0.28084 (16)	0.38240 (19)	0.0242 (5)	
C16	−0.0634 (2)	0.30004 (17)	0.4409 (2)	0.0247 (5)	
H16	−0.1475	0.2748	0.4125	0.030*	
C17	−0.0413 (2)	0.35727 (17)	0.54222 (19)	0.0225 (5)	
H17	−0.1114	0.3702	0.5820	0.027*	

### Atomic displacement parameters (Å^2^)

**Table:**

	*U*^11^	*U*^22^	*U*^33^	*U*^12^	*U*^13^	*U*^23^
Br	0.04619 (18)	0.02724 (15)	0.02419 (14)	−0.00004 (12)	−0.00363 (11)	−0.00854 (10)
O	0.0210 (8)	0.0264 (8)	0.0189 (7)	−0.0031 (7)	0.0056 (6)	−0.0072 (6)
N1	0.0236 (10)	0.0210 (10)	0.0190 (9)	−0.0032 (8)	0.0061 (8)	0.0008 (8)
N2	0.0307 (11)	0.0202 (10)	0.0295 (10)	−0.0027 (8)	0.0105 (9)	0.0003 (8)
N3	0.0287 (11)	0.0280 (11)	0.0247 (10)	0.0014 (9)	0.0107 (8)	0.0027 (8)
C1	0.0263 (12)	0.0263 (13)	0.0274 (12)	0.0006 (10)	0.0102 (10)	0.0078 (10)
C2	0.0322 (13)	0.0219 (12)	0.0222 (11)	0.0022 (10)	0.0082 (10)	0.0008 (9)
C3	0.0273 (12)	0.0274 (12)	0.0143 (10)	−0.0054 (10)	0.0040 (9)	−0.0010 (9)
C4	0.0250 (12)	0.0220 (11)	0.0165 (10)	−0.0026 (9)	0.0059 (9)	−0.0032 (9)
C5	0.0196 (11)	0.0245 (12)	0.0166 (10)	−0.0050 (9)	0.0026 (9)	−0.0037 (9)
C6	0.0304 (13)	0.0241 (12)	0.0247 (11)	0.0010 (10)	0.0047 (10)	−0.0028 (10)
C7	0.0306 (13)	0.0290 (13)	0.0261 (12)	−0.0018 (11)	0.0002 (10)	0.0080 (10)
C8	0.0322 (14)	0.0404 (15)	0.0207 (11)	−0.0049 (12)	0.0084 (10)	0.0043 (11)
C9	0.0379 (15)	0.0354 (14)	0.0270 (12)	0.0019 (12)	0.0150 (11)	−0.0017 (11)
C10	0.0315 (13)	0.0244 (12)	0.0247 (11)	−0.0008 (10)	0.0097 (10)	0.0010 (10)
C11	0.0217 (11)	0.0250 (12)	0.0197 (11)	−0.0049 (9)	0.0047 (9)	−0.0017 (9)
C12	0.0251 (12)	0.0189 (11)	0.0156 (10)	0.0004 (9)	0.0033 (9)	0.0027 (8)
C13	0.0232 (12)	0.0238 (12)	0.0196 (10)	−0.0020 (9)	0.0018 (9)	−0.0013 (9)
C14	0.0276 (12)	0.0247 (12)	0.0201 (11)	0.0017 (10)	0.0053 (10)	0.0003 (9)
C15	0.0345 (13)	0.0185 (11)	0.0176 (10)	−0.0005 (10)	−0.0009 (10)	0.0012 (9)
C16	0.0244 (12)	0.0233 (12)	0.0236 (11)	−0.0056 (10)	−0.0034 (9)	0.0044 (10)
C17	0.0217 (12)	0.0233 (12)	0.0229 (11)	0.0000 (9)	0.0048 (9)	0.0042 (9)

### Geometric parameters (Å, °)

**Table:**

Br—C15	1.902 (2)	C1—H1	0.9300
O—C4	1.417 (3)	C2—H2	0.9300
O—C11	1.426 (3)	C3—C4	1.523 (3)
N1—C2	1.336 (3)	C3—H3A	0.9700
N1—N2	1.363 (3)	C3—H3B	0.9700
N1—C3	1.456 (3)	C4—C5	1.517 (3)
N2—C1	1.323 (3)	C4—H4	0.9800
N3—C2	1.327 (3)	C5—C10	1.387 (3)
C12—C13	1.387 (3)	C6—C5	1.386 (3)
C12—C11	1.507 (3)	C6—C7	1.390 (3)
C13—C14	1.393 (3)	C6—H6	0.9300
C13—H13	0.9300	C7—H7	0.9300
C14—H14	0.9300	C8—C7	1.379 (4)
C15—C14	1.381 (3)	C8—C9	1.385 (4)
C15—C16	1.385 (3)	C8—H8	0.9300
C16—C17	1.384 (3)	C9—H9	0.9300
C16—H16	0.9300	C10—C9	1.385 (3)
C17—C12	1.394 (3)	C10—H10	0.9300
C17—H17	0.9300	C11—H11A	0.9700
C1—N3	1.356 (3)	C11—H11B	0.9700
			
C4—O—C11	113.20 (16)	C7—C8—C9	119.6 (2)
C2—N1—N2	109.63 (18)	C7—C8—H8	120.2
C2—N1—C3	129.13 (19)	C9—C8—H8	120.2
N2—N1—C3	120.95 (18)	C8—C9—C10	120.2 (2)
C1—N2—N1	101.85 (19)	C8—C9—H9	119.9
C2—N3—C1	101.93 (19)	C10—C9—H9	119.9
N2—C1—N3	115.7 (2)	C9—C10—C5	120.4 (2)
N2—C1—H1	122.2	C9—C10—H10	119.8
N3—C1—H1	122.2	C5—C10—H10	119.8
N3—C2—N1	110.9 (2)	O—C11—C12	109.32 (18)
N3—C2—H2	124.5	O—C11—H11A	109.8
N1—C2—H2	124.5	C12—C11—H11A	109.8
N1—C3—C4	112.24 (18)	O—C11—H11B	109.8
N1—C3—H3A	109.2	C12—C11—H11B	109.8
C4—C3—H3A	109.2	H11A—C11—H11B	108.3
N1—C3—H3B	109.2	C13—C12—C17	118.9 (2)
C4—C3—H3B	109.2	C13—C12—C11	122.1 (2)
H3A—C3—H3B	107.9	C17—C12—C11	118.9 (2)
O—C4—C5	113.84 (18)	C12—C13—C14	120.6 (2)
O—C4—C3	105.84 (17)	C12—C13—H13	119.7
C5—C4—C3	109.14 (18)	C14—C13—H13	119.7
O—C4—H4	109.3	C15—C14—C13	119.3 (2)
C5—C4—H4	109.3	C15—C14—H14	120.3
C3—C4—H4	109.3	C13—C14—H14	120.3
C6—C5—C10	119.3 (2)	C14—C15—C16	121.0 (2)
C6—C5—C4	121.1 (2)	C14—C15—Br	118.98 (18)
C10—C5—C4	119.6 (2)	C16—C15—Br	119.97 (18)
C5—C6—C7	120.2 (2)	C17—C16—C15	119.1 (2)
C5—C6—H6	119.9	C17—C16—H16	120.4
C7—C6—H6	119.9	C15—C16—H16	120.4
C8—C7—C6	120.3 (2)	C16—C17—C12	121.0 (2)
C8—C7—H7	119.8	C16—C17—H17	119.5
C6—C7—H7	119.8	C12—C17—H17	119.5
			
C11—O—C4—C5	65.5 (2)	C4—C5—C10—C9	−176.2 (2)
C11—O—C4—C3	−174.60 (17)	C7—C6—C5—C10	−0.2 (3)
C4—O—C11—C12	168.62 (17)	C7—C6—C5—C4	177.1 (2)
C2—N1—N2—C1	−0.5 (2)	C5—C6—C7—C8	−0.5 (4)
C3—N1—N2—C1	−174.8 (2)	C9—C8—C7—C6	0.3 (4)
N2—N1—C2—N3	0.4 (3)	C7—C8—C9—C10	0.6 (4)
C3—N1—C2—N3	174.1 (2)	C5—C10—C9—C8	−1.3 (4)
N2—N1—C3—C4	83.5 (2)	C13—C12—C11—O	−1.9 (3)
C2—N1—C3—C4	−89.6 (3)	C17—C12—C11—O	179.24 (19)
N1—N2—C1—N3	0.4 (3)	C11—C12—C13—C14	−179.4 (2)
C1—N3—C2—N1	−0.1 (3)	C17—C12—C13—C14	−0.6 (3)
N2—C1—N3—C2	−0.2 (3)	C12—C13—C14—C15	0.5 (3)
N1—C3—C4—O	58.0 (2)	Br—C15—C14—C13	178.28 (17)
N1—C3—C4—C5	−179.15 (18)	C16—C15—C14—C13	0.0 (3)
O—C4—C5—C6	38.9 (3)	Br—C15—C16—C17	−178.62 (17)
O—C4—C5—C10	−143.9 (2)	C14—C15—C16—C17	−0.4 (3)
C3—C4—C5—C6	−79.1 (3)	C15—C16—C17—C12	0.2 (3)
C3—C4—C5—C10	98.1 (2)	C16—C17—C12—C11	179.1 (2)
C6—C5—C10—C9	1.1 (4)	C16—C17—C12—C13	0.3 (3)

### Hydrogen-bond geometry (Å, °)

**Table:**

*D*—H···*A*	*D*—H	H···*A*	*D*···*A*	*D*—H···*A*
C13—H13···O	0.93	2.37	2.723 (3)	102
C11—H11A···Cg3^i^	0.97	2.84	3.687 (2)	147

Symmetry codes: (i) −*x*+1, −*y*, −*z*.
